# Gene-specific repair of Pt/DNA lesions and induction of apoptosis by the oral platinum drug JM216 in three human ovarian carcinoma cell lines sensitive and resistant to cisplatin

**DOI:** 10.1038/sj.bjc.6694381

**Published:** 1999-12

**Authors:** C F O’Neill, B Koberle, J R W Masters, L R Kelland

**Affiliations:** Cancer Research Campaign Centre for Cancer Therapeutics, The Institute of Cancer Research, 15 Cotswold Road, Sutton, Surrey SM2 5NG, UK; University College London, Institute of Urology, 67 Riding House Street, London W1P 7PN, UK

**Keywords:** JM216, cisplatin, gene-specific repair, apoptosis

## Abstract

JM216, an oral platinum drug entering into phase III clinical trial, exhibited comparable cytotoxicity to cisplatin in three human ovarian carcinoma cell lines: the sensitive (CH1), acquired resistant (CH1cisR) and intrinsically resistant (SKOV-3). Platinum accumulation and binding to DNA were similar in each of the three cell lines at equimolar doses, indicating that the resistant cell lines could tolerate higher intracellular platinum levels and platinum bound to DNA at IC_50_ concentrations of drug. Comparison with cisplatin demonstrated that intracellular platinum levels were marginally higher with JM216, but that platinum binding to DNA was similar for the two drugs in each of the cell lines. Each of the cell lines exhibited an ability to repair JM216 induced platinum/DNA lesions in the N-*ras* gene (gene-specific repair) at equitoxic concentrations of drug. However, this occurred to a greater extent in the two resistant cell lines such that by 24 h the CH1cisR and SKOV-3 had removed 72% and 67% respectively compared with approximately 32% for the CH1. Reduced gene-specific repair capacity in CH1 cells was also seen following incubation with 25 μM (or 5 μM – 2 × IC_50_) cisplatin, whereas the CH1cisR and SKOV-3 cell lines were repair proficient. JM216 induced apoptosis in the three cell lines following a 2h incubation with 2 × the IC_50_ of drug. Fluorescent microscopy of cells stained with propidium iodide showed that the detached cell population displayed typical apoptotic nuclei. Furthermore, field inversion gel electrophoresis demonstrated the presence of DNA fragments approximately 23–50 kb in size, indicative of apoptosis, in the detached cells. JM216 induced an S phase slow down in each of the three cell lines accompanied by a G2 block in the CH1 pair. Incubation with this concentration of JM216 also resulted in the induction of p53 in the CH1 and CH1cisR. These studies suggest that the relative sensitivity of the CH1 cell line to cisplatin and JM216 is at least partly attributable to a deficiency in gene-specific repair. The oral platinum drug, JM216, exerts its cytotoxic effects through the induction of apoptosis following a slow-down in S phase in both the sensitive and resistant lines. © 1999 Cancer Research Campaign


*British Journal of Cancer* (1999) **81**(8), 1294–1303
© 1999 Cancer Research Campaign
Article no. bjoc.1999.0844

### Gene-specific repair of Pt/DNA lesions and induction of apoptosis by the oral platinum drug JM216 in three human ovarian carcinoma cell lines sensitive and resistant to cisplatin

**CF O'Neill[1], B Koberle[2], JRW Masters[2] and LR Kelland[1]**

[1]Cancer Research Campaign Centre for Cancer Therapeutics, The Institute of Cancer Research, 15 Cotswold Road, Sutton, Surrey SM2 5NG, UK;
[2]University College London, Institute of Urology, 67 Riding House Street, London W1P 7PN, UK

**Summary** JM216, an oral platinum drug entering into phase III clinical trial, exhibited comparable cytotoxicity to cisplatin in three human ovarian carcinoma cell lines: the sensitive (CH1), acquired resistant (CH1cisR) and intrinsically resistant (SKOV-3). Platinum accumulation and binding to DNA were similar in each of the three cell lines at equimolar doses, indicating that the resistant cell lines could tolerate higher intracellular platinum levels and platinum bound to DNA at $IC_{50}$ concentrations of drug. Comparison with cisplatin demonstrated that intracellular platinum levels were marginally higher with JM216, but that platinum binding to DNA was similar for the two drugs in each of the cell lines. Each of the cell lines exhibited an ability to repair JM216 induced platinum/DNA lesions in the N-*ras* gene (gene-specific repair) at equitoxic concentrations of drug. However, this occurred to a greater extent in the two resistant cell lines such that by 24 h the CH1cisR and SKOV-3 had removed 72% and 67% respectively compared with approximately 32% for the CH1. Reduced gene-specific repair capacity in CH1 cells was also seen following incubation with 25 μM (or 5 μM – $2 \times IC_{50}$) cisplatin, whereas the CH1cisR and SKOV-3 cell lines were repair proficient. JM216 induced apoptosis in the three cell lines following a 2h incubation with $2 \times$ the $IC_{50}$ of drug. Fluorescent microscopy of cells stained with propidium iodide showed that the detached cell population displayed typical apoptotic nuclei. Furthermore, field inversion gel electrophoresis demonstrated the presence of DNA fragments approximately 23–50 kb in size, indicative of apoptosis, in the detached cells. JM216 induced an S phase slow down in each of the three cell lines accompanied by a G2 block in the CH1 pair. Incubation with this concentration of JM216 also resulted in the induction of p53 in the CH1 and CH1cisR. These studies suggest that the relative sensitivity of the CH1 cell line to cisplatin and JM216 is at least partly attributable to a deficiency in gene-specific repair. The oral platinum drug, JM216, exerts its cytotoxic effects through the induction of apoptosis following a slow-down in S phase in both the sensitive and resistant lines. © 1999 Cancer Research Campaign

**Keywords**: JM216; cisplatin; gene-specific repair; apoptosis

JM216 (bis acetato ammine dichlorocyclohexylamine platinum IV) is an orally administered platinum (Pt) compound which has been evaluated in phase II clinical trials against small-cell lung cancer, non-small-cell lung cancer, prostate and ovarian cancer and is currently undergoing phase III clinical evaluation (McKeage et al, 1995). It was selected as the lead compound of a series of ammine/amine Pt (IV) dicarboxylates, designed to show improved lipophilicity and gastrointestinal (GI) stability in an effort to overcome the limited GI absorption observed with cisplatin and carboplatin (Giandomenico et al, 1991; Harrap et al, 1991).

In preclinical studies, JM216 exhibited activity comparable to that of cisplatin against a panel of in vitro human ovarian carcinoma cell (HOC) lines (Kelland et al, 1993), lung cancer (Twentyman et al, 1992) and human cervical squamous cells (Mellish et al, 1993), and exhibited non-cross-resistance to cisplatin in a panel of six pairs of acquired resistant and parent human tumour lines, notably where resistance was mediated through reduced Pt uptake (Kelland et al, 1992*a*, 1993).

JM216 undergoes extensive biotransformation in vitro and in vivo and several metabolites, each of which show growth inhibitory activity, have been identified (Poon et al, 1995; Raynaud et al, 1996*a*, 1996*b*). The metabolic profile of JM216 in vitro was found to be dependent upon glutathione levels (Raynaud et al, 1996*b*). For example, in SKOV-3 cells with twice the glutathione content level of CH1, glutathione adducts accounted for 89% of total Pt, compared with 24% in the CH1.

Our previous studies with cisplatin in the CH1 cell line, its acquired resistant variant, CH1cisR and the intrinsically resistant SKOV-3 cell line, showed that there was no difference in platinum accumulation, Pt binding to DNA or global removal of Pt adducts from DNA. However, resistance could be related to reduced formation of interstrand cross-links (ISCs) (O'Neill et al, 1995). In recent years the importance of gene-specific repair in platinum resistance has been well documented (Zhen et al, 1992; Johnson et al, 1994; Koberle et al, 1996, 1997; Petersen et al, 1996). For example, it was demonstrated that Pt/DNA lesions induced in the *DHFR* gene were repaired in the Pt-resistant L1210 cells compared with the parental line when no difference was observed in the overall removal of total Pt (Petersen et al, 1996).

*Received 26 February 1999*
*Revised 7 June 1999*
*Accepted 11 June 1999*

*Correspondence to*: CF O'Neill

Furthermore, the sensitivity of testis tumour cell lines to cisplatin corresponded with a lack of gene-specific repair of lesions induced in the N-*ras* gene compared with repair competent bladder cell lines, more resistant to cisplatin (Koberle et al, 1996, 1997).

Cisplatin and two novel Pt compounds, JM149 and JM335, were shown to induce apoptosis in these cell lines (Ormerod et al, 1996; O'Neill et al, 1996). The protein p53 is known to induce either a G1 arrest or trigger apoptosis following exposure to genotoxic agents including ionizing radiation and cisplatin (Kastan et al, 1991; Lowe et al, 1993, 1994; Hainaut, 1995). These cell lines have been shown to differ in their p53 status, the CH1 and CH1cisR being wild-type for p53 and the SKOV-3, null (Yaginuma and Westphal, 1992; Pestell et al, 1998).

The aim of this study was to determine the cellular pharmacology of JM216 in the CH1, CH1cisR and SKOV-3 cell lines. The variables studied were DNA binding and removal at the level of a portion of the N-*ras* gene, apoptosis, cell cycle effects and the effects on the induction of p53.

### MATERIALS AND METHODS

### Drugs and chemicals

Cisplatin and JM216 were synthesized and obtained from The Johnson Matthey Technology Centre (Reading, Berkshire, UK). Both drugs were dissolved in sterile 0.9% sodium chloride (NaCl) and sterile filtered prior to use. Platinum atomic absorption standard was purchased from Aldrich Chemical Company (Gillingham, Dorset, UK). Phenol (Ultrapure), Dulbecco's modified Eagles' medium (DMEM), trypsin (0.25%) EDTA and cell culture supplements, were purchased from Gibco-BRL (Uxbridge, Middlesex, UK). Novex precast polyacrylamide electrophoresis (PAGE) gels, running and transfer buffers, and nitrocellulose membranes were purchased from R&D systems (Abingdon, Oxford, UK). Monoclonal antibodies and Pierce BCA protein assay reagent kit, were bought from Santa Cruz Biotechnology (Santa Cruz, CA, USA) and Pierce (Rockford, IL, USA) respectively. Forward and reverse primers for QPCR, Buffer IV, NaCl and *Taq* polymerase were purchased from Oswell DNA Service (Southampton, UK). Radiochemicals and Rainbow standard markers, enhanced chemiluminescence reagents (ECL) and Hyper film-ECL were obtained from Amersham International plc (Aylesbury, Bucks, UK) and all other reagent chemicals and enzymes were purchased from Sigma Chemical Co. Ltd (Poole, Dorset, UK) unless otherwise stated.

### Cell culture

Three human ovarian carcinoma cell lines; the CH1 parent, CH1cisR (with acquired resistance to cisplatin) and the intrinsically resistant SKOV-3, were used in these experiments (Fogh et al, 1977; Hills et al, 1989; Kelland et al, 1992*b*). Cells were cultured as monolayers in DMEM supplemented with 10% heat-inactivated fetal calf serum (FCS), MEM non-essential amino acids, 2 mM glutamine and 0.4 μg $ml^{-1}$ hydrocortisone. Under these conditions CH1 cell lines exhibited a doubling time of around 17 h while that of the SKOV-3 cell line was approximately 22 h. Cells in culture were periodically tested and found to be free of *Mycoplasma* using the MycoTect kit (Life Science Technologies).

### Growth inhibition

The effects of JM216 on cell proliferation were measured using the sulphorhodamine B (SRB) assay (Kelland et al, 1992*a*). Briefly, cells were incubated with a range of drug concentrations (in quadruplicate) for 2 h followed by 96 h in drug-free medium. At the end of the 96h period, medium was removed, cells were fixed for 30 min at 4°C with 10% w/v trichloroacetic acid (TCA), and then washed four times with water. The plates were then air-dried and a 0.4% SRB solution in 1% acetic acid added for 10–15 min. The plates were then washed four times with 1% acetic acid and air dried overnight. The SRB was solubilized with 100 μl of 10 mM Tris and the resultant colour intensity quantitated at 540 nm using a Titertek Multiscan MCC/340 (Flow Laboratories). The $IC_{50}$ was determined using Titersoft computer software to determine the concentration of drug, which reduced absorbance by 50%. In these experiments the 2 h $IC_{50}$ obtained for the CH1 (3.8 μM) and SKOV-3 (40.5 μM) were similar to those published in other studies (Mellish et al, 1994; Raynaud et al, 1996), the SKOV-3 being approximately 10.6-fold more resistant to JM216.

### Platinum accumulation

Intracellular (Pt) levels were measured following a 2h exposure to 10 μM, 25 μM, 50 μM and 100 μM concentrations of drug. Cells at approximately $2 \times 10^6$ were washed twice with 10ml volumes of ice-cold phosphate-buffered saline (PBS-4°C) and the monolayer scraped into 2 ml ice-cold PBS. The cell suspensions were then sonicated on ice ($2 \times 30$ s pulses at an amplitude of 20 μm) using a Soniprep 150 (Fisons, Loughborough, UK). Pt content was determined by flameless atomic absorption spectroscopy (FAAS) using a Perkin-Elmer 1100B/HGA 700 (Beaconsfield, Bucks, UK). Aliquots of cell sonicate were automatically injected into the graphite furnace and the ng Pt $ml^{-1}$ of sample measured by comparison with Pt standards 40–200 ng $ml^{-1}$, dissolved in 0.2% nitric acid. Under these conditions the limit of detection was between 5 and 10 ng Pt $ml^{-1}$. Protein content was measured by the method of Lowry et al (1951), where 100 μl of cell sonicate was hydrolysed overnight in a final volume of 1 ml 1 N sodium hydroxide before assay and results were expressed as nmol Pt $mg^{-1}$ protein.

### Determination of Pt bound to DNA

Cells ($5 \times 10^7$) were incubated with a range of drug concentrations (10–100 μM) for 2 h and removed from the monolayer by incubation with trypsin–EDTA (0.25%) for 5 min and DNA extracted. Briefly, the trypsin was neutralized with an equal volume of supplemented DMEM, cells were harvested by centrifugation, washed twice with ice-cold PBS, suspended in 2.5 ml of lysing solution (10 mM Tris, 10 mM EDTA, 150 mM sodium chloride (NaCl), sodium dodecyl sulphate (SDS) 0.4%, 1 mg $ml^{-1}$ proteinase K) and placed at 65°C for 15 min. Residual protein was removed with addition of an equal volume of phenol reagent (500 g phenol, 75 ml M-cresol, 55 ml water, 0.5 g hydroxyquinoline) (Kirby and Cook, 1967). Following centrifugation at 2000 ***g*** for 20 min the aqueous phase was removed, sodium acetate added (0.3 M final concentration) and nucleic acids precipitated by the addition of 2.5 volumes of 100% ethanol. The precipitates were washed twice in 80% ethanol by centrifugation, the resultant pellets were dissolved in 5 ml of 10 mM Tris/0.1 mM EDTA pH 7.7

*© 1999 Cancer Research Campaign* 

and incubated with heat inactivated RNAase (0.1 mg $ml^{-1}$ final concentration) at 37°C for 1 h. The solutions were re-extracted with phenol reagent and DNA precipitated as above. Dried DNA pellets were dissolved overnight at 37°C in 0.2% nitric acid and platinum content measured by FAAS, DNA content was measured using the Burton assay (1956) and results were expressed as pmoles of Pt per mg of DNA.

### Measurement of Pt/DNA lesions using quantitative PCR

Formation and removal of platinum induced lesions was measured in the N-*ras* gene following the method outlined by Grimaldi et al (1994) and Koberle et al (1996). Cells in mid-log phase were incubated for 5 h with drug in order to optimize detection of lesions (B Koberle, unpublished observations) and collected as above immediately after removal of drug or following a 24 h incubation in drug-free medium. Approximately $10^6$ cells were lysed in a 2 ml Eppendorf in 340 µl of lysis solution (400 mM Tris pH 8, 60 mM EDTA, 150 mM NaCl, 1% w/v SDS) plus 100 µl 5 M sodium perchlorate, by mixing at room temperature for 20 min and then at 65°C for a further 20 min. Then, 580 µl of chloroform was added to the lysate, the solution mixed on a rotary mixer for 20 min at room temperature followed by centrifugation in a microfuge at top speed for 10 min. Following centrifugation, the upper layer was aliquoted into a new Eppendorf and nuclear material precipitated in 2 volumes of absolute ethanol. The precipitate was washed twice in 70% ethanol, dried in an incubator overnight at 37°C and dissolved in 400 µl water. Polymerase chain reaction (PCR) was performed using 25 µl of sample in a final volume of 100 µl containing: 2 units (0.2 µl) of *Taq* polymerase (red hot), 4.8 µl of a dATP, dCTP, dGTP, dTTP mix (120 mM each), 3 µl (0.75 mM) magnesium chloride ($MgCl^2$) 10 µl Buffer IV, 5 µl DMSO, 1 µl each of 3′ and 5′ primer sequence, 49.9 µl water and 1 µCi $^{\alpha\text{-}32}$P-dCTP. The amplification of the N-*ras* fragment was performed using the forward primer 5′-GCC TGG TTA CTG TGT CCT GT-3 and the reverse primer 5′-GCC AGC CAC ATC TAC AGT AC-3′. PCR was carried out using a Perkin-Elmer 480 thermal cycler, an initial 2 min denaturation step at 94°C was followed by 25 cycles of 94°C, 55°C and 72°C for 1 min each, with a final incubation of 4 min at 72°C at the end of the cycling. These conditions were chosen to ensure that the PCR reaction was still in the exponential phase when the reaction was stopped.

Aliquots of 40 µl of reaction mixture were precipitated with 1 ml ice-cold 5% w/v trichloroacetic acid-20 mM tetrasodium pyrophosphate (TCA). The precipitate was captured on Whatman GF/C filters with unincorporated $^{\alpha\text{-}32}$-P-dCTP washed through with 10 ml of the TCA solution followed by 10 ml of absolute ethanol. The filters were placed in scintillation fluid and the PCR product quantified by measuring counts per minute (cpm). DNA content was measured using the Burton assay and cpm $\mu g^{-1}$ DNA calculated with these results used to calculate the lesion index for each sample.

The lesion index was calculated using the formula-ln Ad/A where A represents cpm from control sample and Ad represents cpm from treated samples. Controls containing 25% and 50% of the control sample were included to ensure that the PCR was quantitative.

### Microscopy

Attached (control) cells harvested by incubation with trypsin and detached cells generated from the CH1, CH1cisR and SKOV-3 cell lines following exposure to 7.6 µM 28 µM and 81 µM JM216 (2 × $IC_{50}$) respectively, were collected, centrifuged and suspended in 1 mg $ml^{-1}$ propidium iodide. Morphology was examined by fluorescent microscopy after a 10 min incubation at room temperature.

### Field inversion gel electrophoresis

Attached and detached cells were collected as above. Approximately 5 × $10^5$ cells were incubated for 1 h at 37°C in 100 µl lysis buffer (200 mM Tris, 100 mM EDTA, 2% SDS) containing 1 mg $ml^{-1}$ proteinase K and RNAase (heat-inactivated) final concentration. Aliquots of cell lysate were added directly to the gel (1% agarose in TAE buffer), and the wells sealed with 1% low melting-point agarose. Field inversion gel electrophoresis (FIGE) was performed with 1 × TAE (40 mM Tris, 20 mM sodium acetate, 1 mM EDTA) using a Bio Rad FIGE Mapper. Horizontal gels were run for 20 h with a forward voltage of 10 V $cm^{-1}$ and reverse of 7 V $cm^{-1}$ with linear ramping T1 = 1 s to T2 = 12 s. The temperature of buffer was controlled to 14°C using a Bio-Rad 1000 'mini chiller'. Sigma Pulse Marker 1 fragments of 0.1–200 kb in size were run with the samples.

### Cell cycle analysis

This was carried out as outlined in Ormerod et al (1996). Briefly, cells were collected by centrifugation and suspended in 200 µl ice-cold PBS and fixed by the addition of 1.8 ml ice-cold 70% ethanol, for at least 30 min on ice. Fixed cells were centrifuged and the pellets suspended in 800 µl PBS, 100 µl (1 mg $ml^{-1}$) RNAase and 100 µl of PI (200 µg $ml^{-1}$) and incubated at 37°C for at least 30 min, then overnight at 4°C. Flow cytometry was carried out on a Coulter Elite equipped with a Spectra-physics argon-ion laser with an output of 200 mW at 488 nm. Typically data from 2 × $10^4$ cells were analysed with forward and orthogonally scattered light and red fluorescence (peak and integrated area) recorded. DNA histograms were generated using WinMDI2.6 Windows Multiple document Interface Flow Cytometry Application (obtained from the Internet), from which cell cycle data was calculated using software employing a Watson algorithm (Ormerod et al, 1987).

### Western blot analysis

CH1 and CH1cisR cells were incubated for 2 h with 7.6 µM and 28 µM JM216 respectively and harvested as above (see measurement of Pt bound to DNA) at 5, 16 and 24 h following removal of drug. Drug additions were staggered so as to facilitate simultaneous collection of all time points. Following two washes in ice-cold PBS the resultant cell pellets from control and treated samples were resuspended in 300 µl of lysis buffer (150 mM NaCl, 50 mM Tris–HCl) to which was added 500 µl of 20 mM phenylmethylsulphonyl fluoride (PMSF), 100 µl sodium orthovanadate (10 mM stock), 100 µl NP40, 100 µl 20% SDS, 2 µl aprotinin (10 mg $ml^{-1}$ stock) and 2 µl leupeptin (10 mg $ml^{-1}$ stock) and incubated on ice for 1 h. Lysis was aided by pipetting using a Gilson P200 pipette. The lysed samples were centrifuged at 12 000 rpm at 4°C for 15 min and the supernatant collected for analysis. Protein content was determined using the Pierce BCA protein detection kit. Samples were diluted with water to ensure that identical concentrations of protein were loaded onto the gel and then diluted 1/2 with Laemmli buffer and incubated at 95°C for 3 min.

© 1999 Cancer Research Campaign

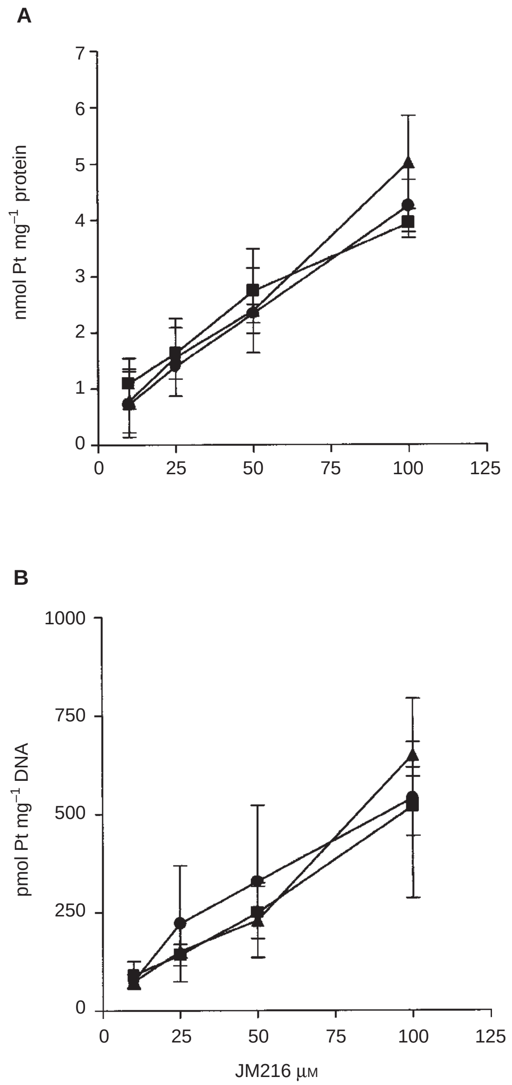

**Figure 1** Pt accumulation (**A**) and Pt bound to DNA (**B**) the CH1 (■), CH1cisR (▲) and SKOV-3 (●) following a 2 h incubation with a range of JM216 concentrations (10–100 μM). Error bars = s.d. where *n* = at least 3

Typically, 50 μg of protein (25-μl sample) and 3 μl of standard rainbow marker were loaded onto 8–16% Novex precast PAGE gels and run at 30 mA per gel for 1 h, then transferred to nitrocellulose membrane at 300 mA for 2 h. The membranes were washed twice with PBS containing 0.1% Tween-20 (PBST), excess liquid was drained off, then the membranes placed in blocking buffer (154 mM NaCl, 10 mM Tris, 0.5% caesin and 0.02% thimerosal, at pH 7.6) and agitated for 1 h. The membranes were subsequently washed in PBST for 5 min and probed for the protein of interest. Following incubation with primary antibody the membranes were washed 3 × 10 min with PBST and incubated with secondary antibody linked with horseradish peroxidase for at least 1 h and then detected with ECL reagents by autoradiography. The primary antibody used for p53 was the DO-1 mouse IgG-2a mouse monoclonal detected with anti-mouse IgG linked to horseradish peroxidase (Amersham).

### Statistical analysis

Where appropriate, statistical significance was tested using a two-tailed Student's *t*-test.

**Table 1** Expected intracellular and Pt bound to DNA levels at $IC_{50}$ concentrations of JM216 calculated from data generated for Figure 1 A and B

| Cell line | nmoles Pt $mg^{-1}$ protein at $IC_{50}$ | pmoles Pt $mg^{-1}$ DNA at $IC_{50}$ |
|---|---|---|
| CH1 | 0.16 | 19.7 |
| CH1cisR | 0.71 | 85.7 |
| SKOV-3 | 1.77 | 234.6 |

**Table 2** Comparison of intracellular Pt levels following 2 h incubation with 50 μM JM216 and cisplatin. Error bars = s.d. where *n* = 3; *n* = 2 for cisplatin Pt $mg^{-1}$ protein

| Cell line | nmoles Pt $mg^{-1}$ protein | | pmoles Pt $mg^{-1}$ DNA | |
|---|---|---|---|---|
| | JM216 | Cisplatin | JM216 | Cisplatin |
| CH1 | 2.7 ± 0.7 | 1.3 | 249 ± 66 | 226 ± 6 |
| CH1cisR | 2.3 ± 0.7 | 1.5 | 229 ± 94 | 174 ± 44 |
| SKOV-3 | 2.3 ± 0.1 | 1.4 | 328 ± 192 | 197 ± 65 |

### RESULTS

### Cell growth inhibition by JM216

The concentration of JM216 required to inhibit growth of CH1cisR cells by 50% ($IC_{50}$) following a 2 h incubation was 14 μM. Thus the resistance factor (Rf) for the CH1cisR was 3.6.

### Platinum accumulation and platinum bound to DNA with JM216

Following incubation with JM216, intracellular Pt levels were similar in all three cell lines and increased with increasing levels of drug (Figure 1A). For example, at 50 μM JM216, intracellular Pt levels were approximately: CH1 2.73 ± 0.7, CH1cisR 2.39 ± 0.7, SKOV-3 2.33 ± 0.1 nmol $mg^{-1}$ protein.

Similarly, there was no cell line-dependent difference in the levels of Pt bound to DNA (Figure 1B), such that at 50 μM JM216 levels of Pt bound to DNA were approximately: CH1 249 ± 66, CH1cisR 229 ± 94, SKOV-3 328 ± 192 pmol $mg^{-1}$ DNA. These results indicate that per mg, the amount Pt bound to DNA was roughly 10% that of protein bound Pt. Furthermore, by calculating the expected levels of Pt at equitoxic levels of drug, it could be demonstrated that the resistant cell lines possessed higher intracellular Pt levels and could tolerate higher levels of Pt bound to DNA (Table 1).

Platinum uptake and Pt/DNA levels were compared in the three cell lines following 50 μM cisplatin and JM216 (Table 2). Intracellular Pt levels were on average 1.8-fold higher with JM216. However, levels of Pt bound to DNA were similar for both drugs.

### Gene-specific repair of Pt/DNA lesions using QPCR

Initially, the levels of Pt/DNA lesions in the N-*ras* gene were measured in CH1, CH1cisR and SKOV-3 cells immediately following a 5 h incubation with 25 μM cisplatin and then at 24 h following the removal of drug, using the technique described by Grimaldi et al (1994) and Koberle et al (1996).

*© 1999 Cancer Research Campaign*

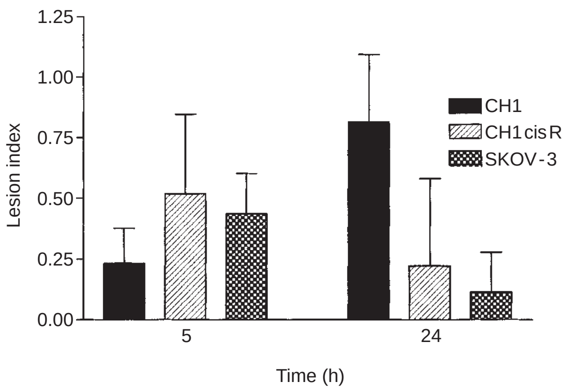

**Figure 2** Measurement of induction and repair of lesions induced in the N-*ras* gene following a 5 h incubation at 25 μM cisplatin. Error bars = s.d. where *n* = at least 3

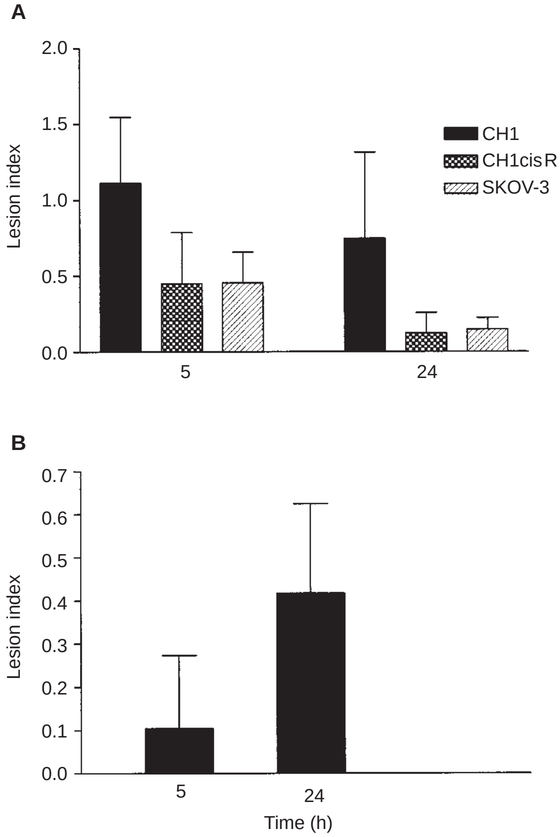

**Figure 3** Measurement of induction and repair of lesions induced in the N-*ras* gene, (**A**) following a 5 h incubation at 2 × the $IC_{50}$ concentration of JM216 (CH1 7.6 μM, CH1cisR 28 μM, SKOV-3 81 μM) for each cell line. (**B**) Following a 5 h incubation with 5 μM cisplatin in the CH1. Error bars = s.d. where n = at least 3

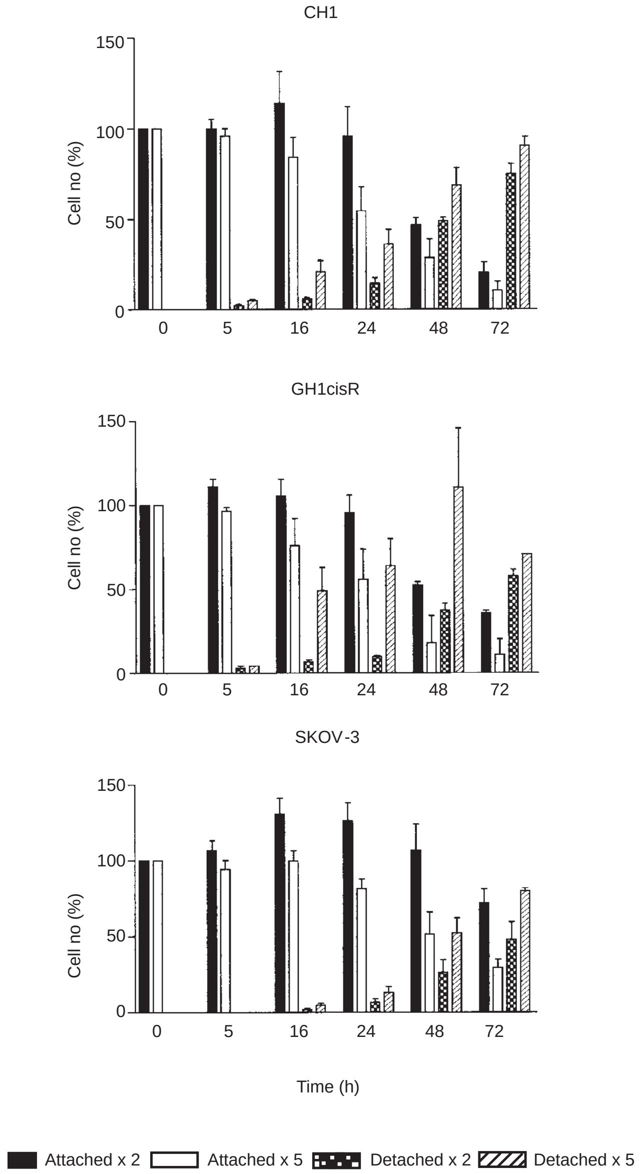

**Figure 4** Measurement of attached and detached cells remaining following exposure to 2 × and 5 × the $IC_{50}$ of JM216 in CH1, CH1cisR, and SKOV-3. Attached cells were expressed as a percentage of cell number at time 0 and detached cells were expressed as a percentage of total cell number (attached + detached) at each time point. Error bars = s.d. where *n* = at least 3

The mean levels of Pt/DNA adducts in the N-*ras* gene were similar in the two resistant cell lines but approximately 50% lower in the CH1-sensitive cell line, though the difference was not quite statistically significant (Figure 2). However, by 24 h following removal of drug, both the resistant cell lines exhibited significantly reduced levels of lesions ($P < 0.05$) in this gene compared with the CH1 cell line. In contrast, the number of lesions increased with time following removal of cisplatin in the CH1 cell line.

Subsequent experiments were carried out with 2 × $IC_{50}$ of JM216, which for each cell line equated to 7.6 μM, 28 μM and 81 μM in the CH1, CH1cisR and SKOV-3 respectively. All the cell lines displayed an ability to repair the lesions induced by JM216 (Figure 3A). Interestingly, while both the CH1cisR and SKOV-3 cell lines contained comparable levels of lesions at 5 h, these levels were approximately 60% lower than in the sensitive CH1 cell line ($P < 0.05$ vs the SKOV-3). By 24 h, the CH1, CH1cisR and SKOV-3 had removed 32%, 72% and 67% respectively, of the initial lesions formed, but the difference between sensitive and resistant cell lines was not quite significant. Furthermore, in the CH1-sensitive cell line, there was a difference of almost 4.8-fold in the lesion index for 7.6 μM JM216 (1.1), compared with 25 μM

© 1999 Cancer Research Campaign

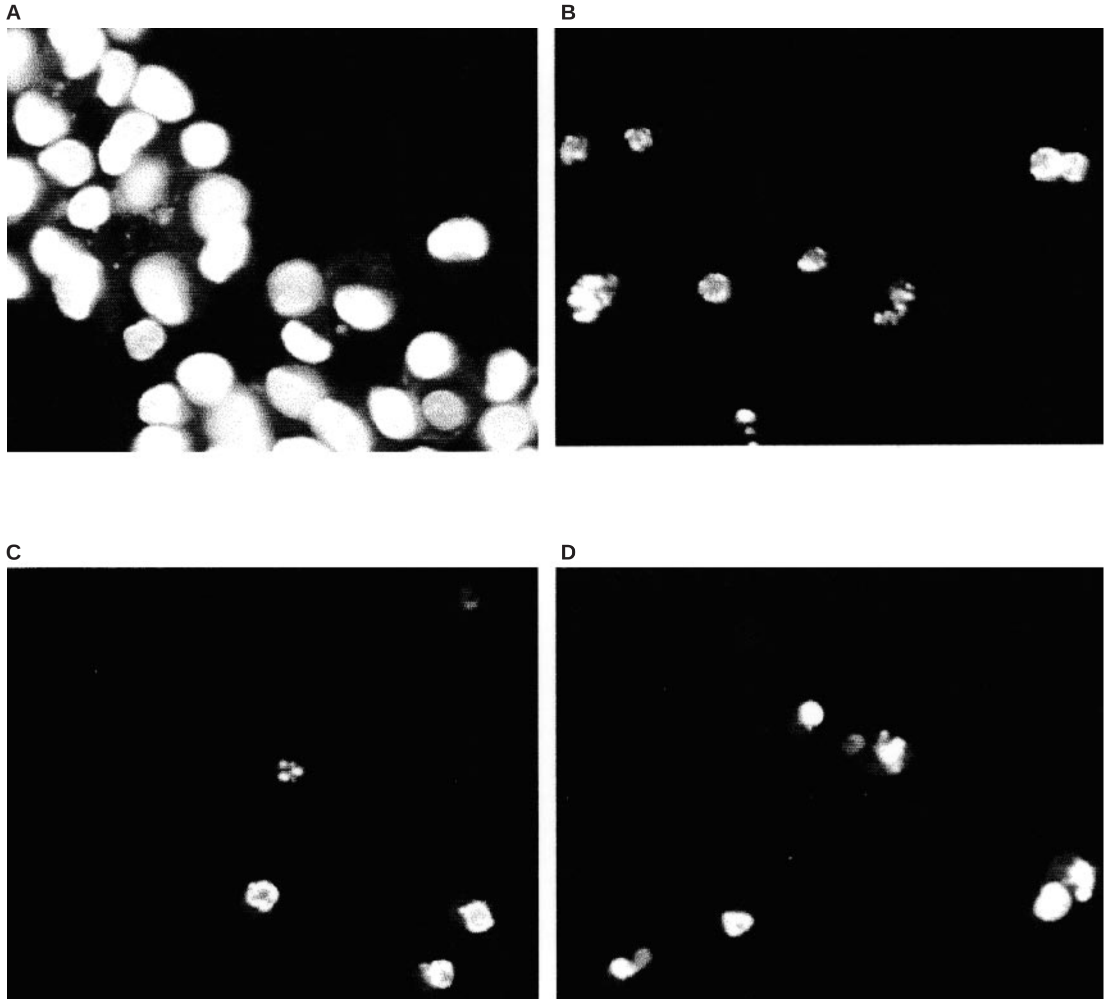

**Figure 5** Fluorescent microscopy showing normal nuclear morphology of CH1 control untreated attached cells (**A**), and apoptotic nuclei of detached cells from CH1 (**B**) 24 h, CH1cisR (**C**) 24 h and SKOV-3 (**D**) 48 h, following exposure to 2 × the $IC_{50}$ of JM216

cisplatin (0.23). The moderate repair of JM216 induced lesions in the CH1-sensitive cell line, albeit following a comparatively low concentration, raised the possibility that minor levels of repair of cisplatin-induced lesions by this cell line may have been masked by the 25 μM concentration used. Thus the experiment was repeated with 5 μM cisplatin which was 2 × $IC_{50}$ for the CH1 (Figure 3B). Initial levels of lesions were low, but detectable, following the 5 h incubation, however, as with the higher concentration of drug, the number of lesions increased over the 24 h drug-free period.

### Cell death induced by JM216

The detachment of cells from the monolayer was measured after a 2 h incubation with 2 × and 5 × the 2 h $IC_{50}$ of JM216. The numbers of attached cells remaining after removal of drug were calculated as a percentage of the starting cell number, whereas detached cells were expressed as a percentage of the total number of cells remaining at each time point (Figure 4A–C) (O'Neill et al, 1996). There was an initial increase in cell number up to 16 h with 2 × $IC_{50}$ of drug in the three cell lines. Thereafter, cells detached in a dose- and time-dependent manner with 50%

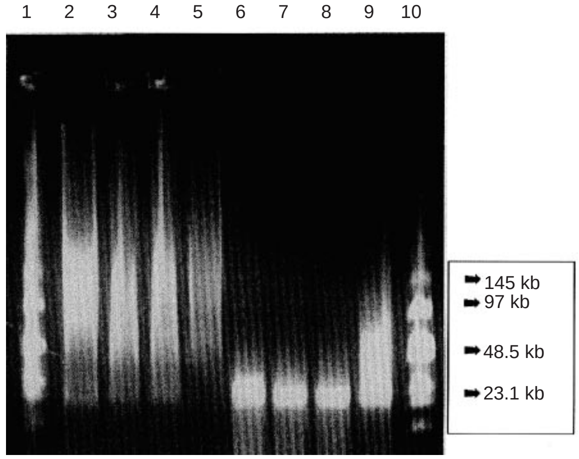

**Figure 6** FIGE of attached and detached cells collected 24 h (CH1 cell lines) and 48 h (SKOV-3) following a 2 h platinum drug exposure. Lanes 1 and 10 are molecular weight markers; lanes 2 and 6 are attached and detached CH1 following exposure to 2 × $IC_{50}$ cisplatin. Lanes 3, 4 and 5 are attached CH1, CH1cisR and SKOV-3 respectively and lanes 7, 8 and 9 their detached counterparts following exposure to 2 × $IC_{50}$ JM216

© 1999 Cancer Research Campaign

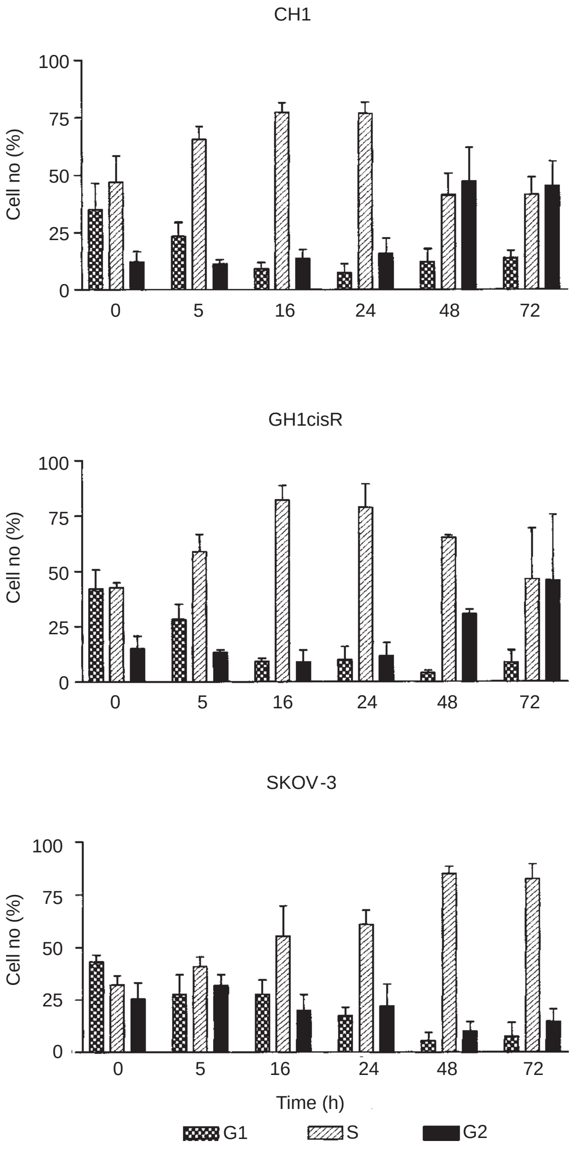

**Figure 7** Cell cycle distribution of CH1, CH1cisR and SKOV-3 with time following exposure to 2 × the $IC_{50}$ of JM216 for 2 h. Error bars = s.d. where $n$ = at least 3

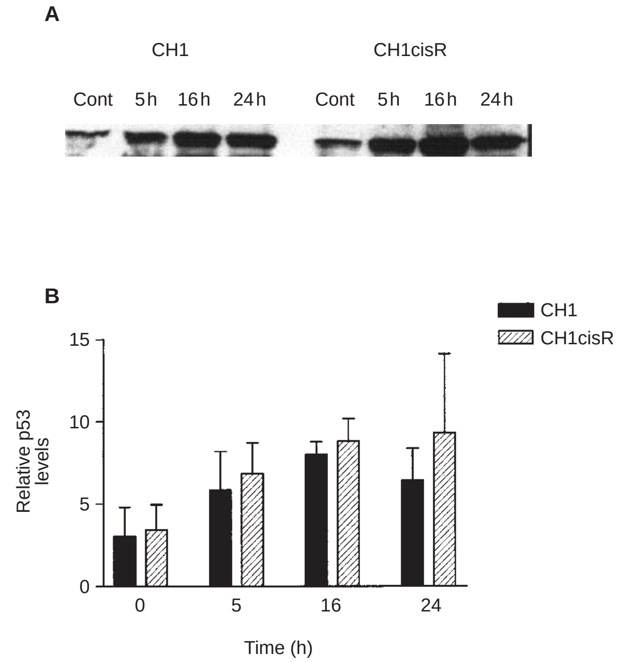

**Figure 8** Analysis of p53 protein levels in the CH1 and CH1cisR following a 2 h incubation with JM216 (7.6 μM CH1, 28 μM CH1cisR). (**A**) Representative Western blot. (**B**) Densitometry analysis of p53 Western blots. Error bar = s.d., $n$ = 3

detachment occurring at 48 h for both CH1 cell lines and 72 h for the SKOV-3 at 2 × the $IC_{50}$.

### Morphology

Detached CH1/CH1cisR and SKOV-3 cells were collected 24 h and 48 h respectively, following incubation with 2 × the $IC_{50}$ of JM216. These cells exhibited apoptotic morphology with compacted and fragmented nuclei when compared with attached control cells where the chromatin was diffuse (Figure 5). Previous studies have shown that the morphology of attached treated cells is similar to that of attached control untreated cells (Ormerod et al, 1994*a*, 1996; O'Neill et al, 1996).

### Field inversion gel electrophoresis

FIGE was carried out on samples prepared from cells collected at 24 h (CH1 cell lines) and 48 h (SKOV-3) following a 2 h exposure to 2 × $IC_{50}$ JM216. Samples collected from CH1 cells 48 h after incubation with 2 × $IC_{50}$ cisplatin, were run as a positive control (Figure 6). Discrete DNA fragments of approximately 23–50 kb, indicative of apoptosis, were detected in the detached cell population from all three cell lines following JM216 and following cisplatin in the CH1. This was absent from the attached cells. However, there was evidence of fragmentation in the attached cell population of both CH1 cell lines running between 90 and 150 kb, which was absent from the SKOV-3 cell line. Earlier studies demonstrated that the DNA from attached control untreated cells does not migrate much beyond the loading point (Ormerod et al, 1994a, 1996)

### Cell cycle analysis

Flow cytometric analysis was carried out from attached cells collected at various time points after incubation with 2 × $IC_{50}$ JM216 (Figure 7). The main cell cycle effect of JM216 was a slow down of traverse through S phase, which culminated in a G2 block occurring from 48 h to 72 h in the CH1 and CH1cisR cell lines following removal of drug. For example, by 48 h there were approximately 37.7% and 47% of cells in S phase and G2 phase respectively in the CH1, compared with 84.8% in S phase and 9.8% in G2 phase for the SKOV-3 ($P < 0.05$). The S phase effect in the SKOV-3 still persisted by 72 h with no evidence of a build up of cells in G2.

### P53 induction

The level of p53 was studied in the CH1 and CH1cisR cell lines before and following a 2 h incubation with 2 × the $IC_{50}$ of JM216 for each cell line. Constitutive levels of p53 were similar in both

 © 1999 Cancer Research Campaign cell lines, but had increased significantly ($P < 0.05$) in each cell line by 16 h after removal of drug, with levels beginning to plateau by 24 h (Figure 8).

### DISCUSSION

This study has addressed the cellular pharmacology of JM216 in one ovarian cell line possessing acquired resistance to cisplatin (CH1cisR), another cell line possessing intrinsic resistance to cisplatin (SKOV-3) and a parental-sensitive cell line (CH1). JM216 exhibited a comparable level of cytotoxicity to that of cisplatin in the CH1, CH1cisR and SKOV-3 human ovarian cancer cell lines. For example, in a previous study, the 2 h $IC_{50}$s for cisplatin were 2.5 μM, 7.5 μM and 33 μM respectively, for the CH1, CH1cisR and SKOV-3, the resistant cell lines being three- and 13.5-fold more resistant to cisplatin (O'Neill et al, 1995). Thus acquired and intrinsic resistance to cisplatin also manifested in resistance to JM216 in these cell lines.

It has been demonstrated that higher levels of GSH in the SKOV-3 (Mistry et al, 1991; Kelland et al, 1992*b*) influence the biotransformation of JM216 and results in differences in the metabolic profile of this drug in vitro when compared with the more sensitive CH1 (Raynaud et al, 1996*b*). Despite these factors, there was no difference in either uptake or the levels of Pt bound to DNA between the three cell lines, across a range of JM216 concentrations (10–100 μM). However, when examined in the context of $IC_{50}$ concentration, these results indicated that, at equitoxic concentrations of JM216, intracellular Pt levels were higher in the CH1cisR and SKOV-3 cell lines, which displayed an ability to tolerate higher levels of Pt bound to DNA. Interestingly, when compared at equimolar concentrations, intracellular levels of Pt for JM216 were marginally higher than for cisplatin in each of the three cell lines. This may have been due to the greater lipophilicity of JM216 enhancing its intracellular accumulation. However, this did not result in higher levels of Pt bound to DNA for this drug compared with cisplatin.

Gene-specific repair of Pt lesions induced by JM216 in the N-*ras* gene occurred in the three cell lines, but to a greater extent in the CH1cisR and SKOV-3. Initial levels of lesions in the resistant cell lines were approximately twofold less than in the CH1, and indeed, were similar to the levels remaining in the N-*ras* gene of the CH1 24 h after removal of drug. This suggested that the CH1 was less capable in repairing these lesions than the two resistant cell lines. A lack of repair capability in the CH1 compared with the CH1cisR and SKOV-3 was demonstrated at a comparatively higher concentration of 25 μM cisplatin. At this concentration, only the resistant cell lines demonstrated repair of lesions in the N-*ras* gene. Moreover, in the CH1, Pt adduct formation continued to rise over the 24-h drug-free period. The reason for this is unclear, previous studies have shown that the formation of inter-strand cross-links (ISC) increase for up to 5 h following an initial 2 h incubation with 25 μM cisplatin, which is more pronounced in the CH1 cell line (O'Neill et al, 1995). It is conceivable that following a 5 h incubation at this concentration of cisplatin, the formation of these ISC is prolonged or that lesions are being converted to strand breaks, both of which might hamper amplification of gene segments by *Taq* polymerase under these experimental conditions.

Being mindful of the possibility that low levels of repair in the CH1 cell line might have been masked by the higher concentrations of cisplatin used, the experiment was repeated at a lower concentration of 5 μM cisplatin or $2 \times IC_{50}$ in this cell line, which was roughly equitoxic with 7.6 μM JM216. The CH1 cell line was still unable to repair the lesions induced by cisplatin at this concentration and, as with the higher dose, the number of lesions continued to increase over the 24-h drug-free period. This difference in the ability to repair the Pt/DNA lesions induced by JM216 in the N-*ras* gene, but not by cisplatin, may indicate that the type of lesion formed by JM216 and/or the recognition of these lesions is different in this cell line. These results are consistent with studies which have demonstrated that gene-specific repair is an important mechanism of resistance in cisplatin resistant cell lines (Zhen et al, 1992; Johnson et al, 1994; Petersen et al, 1996).

JM 216 induced apoptosis in the CH1, CH1cisR and SKOV-3 cell lines at 2 × the $IC_{50}$ concentration of drug for each cell line. This compliments earlier studies where cisplatin and two novel *cis* and *trans* Pt compounds, JM149 and JM335 were shown to induce apoptosis in these cell lines (Ormerod et al, 1996; O'Neill et al, 1996). Cells detached in a dose- and time-dependent manner after removal of drug, with 50% detachment occurring at 48 h for both CH1 cell lines and 72 h for the SKOV-3, at 2 × the $IC_{50}$. The difference in the rate of cell death may have been due to the difference in doubling times for the CH1 pair (17 h) and the SKOV-3 (22 h). Apoptotic nuclei were detected by fluorescence microscopy following incubation with PI and the characteristic 23–50 kb DNA fragment associated with apoptosis was detected by FIGE (Oberhammer et al, 1993; Ormerod et al, 1994*a*, 1996; O'Neill et al, 1996).

Flow cytometric analysis revealed that a slow down in S phase transit was a prominent cell cycle effect of JM216 in the three cell lines. This was accompanied by a marked accumulation of cells in G2 in the CH1 and CH1cisR cell lines by 72 h. However, with the SKOV-3 cell line, the majority of cells were still in S phase with no evidence of a build up of cells in G2 at this time point. Similar cell cycle perturbations were observed with cisplatin in each cell line (Ormerod et al, 1996) and in the CH1 cell line with JM149 and JM335 (O'Neill et al, 1996), and are consistent with the observations of other authors (Vaisman et al, 1997; Zaffaroni et al, 1998). Taken together these and other studies indicate that accumulation of cells in S phase, is a general cell cycle effect of platinum drugs, which depending on the cell type, is associated with G2 arrest (Ormerod et al, 1994*b*, 1996; O'Neill et al, 1996; Vaisman et al, 1997; Zaffaroni et al, 1998). In the CH1 cell lines, accumulation of cells in G2 coincided with the point at which significant apoptosis was occurring. It has been suggested that G2 arrest facilitates repair of DNA damage prior to mitosis and depending on the success of repair or extent of DNA damage, cells emerging from G2 either begin to cycle normally or engage apoptosis (Sorenson and Eastman, 1988; Ormerod et al, 1994*b*). Our data suggests that the latter may be occurring in the CH1 cell lines, despite the repair of Pt lesions induced by JM216.

Recent evidence has shown that the induction of transfected wild-type p53 at permissive temperatures following irradiation, produced a G2 block in H1229 NSCLC cells (Winters et al, 1998). It is possible then, that the generation of a G2 block by JM216 in the CH1 cell lines could be associated with the induction of p53 by this drug as both the CH1 and CH1cisR are wild-type for p53 (M Walton, personal communication; Pestell et al, 1998), levels of which, increased following exposure to JM216. The work of Pestell et al, demonstrated that across a panel of human ovarian carcinoma cell lines, the expression of wild-type p53 corresponded with sensitivity to ionizing radiation and cisplatin. This is

© 1999 Cancer Research Campaign in agreement with the work of others who have shown that functional status of p53 is thought to be an important determinant of susceptibility to apoptosis (McIlwrath et al, 1994; Perego et al, 1996). Our data showing the induction of p53 at concentrations that induced apoptosis, adds further weight to the evidence that this process may be p53-dependent in the CH1 cell lines.

In conclusion, JM216, in common with other Pt drugs, induced apoptosis in the CH1, CH1cisR and SKOV-3 cell lines. Apoptosis was accompanied by a slow down of passage of cells through S phase in each cell line, and in the CH1 pair by a G2 block. Increased p53 protein levels were measured at concentrations of JM216 that induced apoptosis. JM216 exhibited cross-resistance with cisplatin in the CH1cisR. Intracellular Pt accumulation and binding to DNA were similar in all three cell lines at equimolar drug concentrations. However, at equitoxic levels of JM216, both resistant cell lines exhibited reduced Pt lesion formation in the N-*ras* gene and enhanced repair of these lesions compared with the CH1. Both resistant cell lines, but not the CH1, repaired lesions induced by cisplatin. Thus resistance to JM216 in these lines may be mediated through increased tolerance to Pt/DNA adducts, enhanced gene-specific repair in both resistant cell lines and in part by the elevated GSH levels in the SKOV-3 cell line. Notably in a CH1 subline possessing acquired resistance to JM216, as in CH1cisR, resistance was attributed to increased DNA repair (Mellish and Kelland, 1994)

### ACKNOWLEDGEMENTS

This study was supported by the Cancer Research Campaign. Thanks are due to Johnson Matthey for the supply of platinum drugs.

### REFERENCES

Burton K (1956) A study of the condition and mechanism of the diphenylamine reaction for the coloroietric estimation of deoxyribonucleic acid. *Biochem J* **62**: 315–323

Fogh J, Wright WC and Lovelass JD (1977) Absence of HeLa cell contamination in 169 cell lines transformed in vitro. *J Natl Cancer Inst* **58**: 209–214

Giandomenico CM, Abrams MJ, Murrer BA, Vollano JF, Barnard CFJ, Harrap KR, Goddard PM, Kelland LR and Morgan SE (1991) Synthesis and reactions of a new class of orally active Pt(IV) antitumour complexes. In: *Platinum and Other Metal Coordination Compounds in Cancer Chemotherapy*, Howell SB (ed), pp. 93–100. Plenum: New York

Grimaldi KA, Bingham JP, Souhami RL and Hartley JA (1994) DNA damage by anti cancer agents: mapping in cells at the subgene level with quantitative PCR. *Analyt Biochem* **222**: 236–242

Hainaut P (1995) The tumour suppresser protein p53: a receptor to genotoxic stress that controls cell growth and survival. *Curr Opin Oncol* **7**: 76–82

Harrap KR, Murrer BA, Giandomenico CM, Morgan SE, Kelland LR, Jones M, Goddard PM and Schurig J (1991) Ammine/amine platinum IV platinum dicarboxylates a novel class of complexes which circumvent intrinsic cisplatin resistance. In: *Platinum and Other Metal Coordination Compounds in Cancer Chemotherapy*, Howell SB (ed), pp. 93–100. Plenum: New York

Hills CA, Kelland LR, Abel G, Siracky J, Wilson AP and Harrap KR (1989) Biological properties of ten human ovarian carcinoma cell lines: calibration in vitro against four platinum complexes. *Br J Cancer* **59**: 527–534

Johnson SW, Perez RP, Godwin AK, Yeung AT, Handel LM, Ozols RF and Hamilton TC (1994) Role of platinum-DNA adduct formation and removal in cisplatin resistance in human ovarian cancer cell lines. *Biochem Pharmacol* **47**: 689–697

Kastan MB, Omyekwere O, Sidransky D, Vogelstein B and Craig RW (1991) Participation of p53 protein in cellular response to DNA damage. *Cancer Res* **51**: 6304–6311

Kelland LR, Murrer BA, Abel G, Giandomenico CM, Mistry P and Harrap KR (1992*a*) Ammine/amine platinum (IV) dicarboxylates: a novel class of platinum complexes exhibiting selective cytotoxicity to intrinsic cisplatin-resistant human ovarian carcinoma cell lines. *Cancer Res* **52**: 822–828

Kelland LR, Mistry P, Abel G, Loh SY, O'Neill CF, Murrer BA and Harrap KR (1992*b*) Mechanism-related circumvention of acquired cis-diamminedichloroplatinum(II) resistance using two pairs of human ovarian carcinoma cell lines by ammine/amine platinum(IV) dicarboxylates. *Cancer Res* **52**: 3857–3864

Kelland LR, Abel G, Mckeage MJ, Jones M, Goddard PM, Valenti M, Murrer BA and Harrap KR (1993) Preclinical antitumour evaluation of bis-acetato-ammine-dichloro-cyclohexylamine platinum (IV): an orally active platinum drug. *Cancer Res* **53**: 2581–2586

Kirby KS and Cook EA (1967) Isolation of deoxyribonucleic acid from mammalian tissue. *Biochem J* **104**: 254–257

Koberle B, Payne J, Grimaldi KA, Hartley JA and Masters JRW (1996) DNA repair in cisplatin sensitive and resistant human cell lines measured in specific genes by quantitative polymerase chain reaction. *J Biochem Pharmacol* **52**: 1729–1734

Koberle B, Grimaldi KA, Sunters A, Hartley JA, Kelland LR and Masters JRW (1997) DNA repair capacity and cisplatin sensitivity of human testis tumour lines. *Int J Cancer* **70**: 551–555

Lowe SW, Ruley HE, Jacks T and Housman DE (1993) P53 dependent apoptosis modulates the cytotoxicity of anti cancer agents. *Cell* **74**: 957–967

Lowe SW, Bodis S, McClatchey A, Remington L, Ruley HE, Ficher DE, Housman DE and Jacks T (1994) P53 status and the efficacy of cancer therapy in vivo. *Science* (Washington DC) **266**: 807–810

Lowry OH, Rosenbrough MT, Farr AL and Randall RJ (1951) Protein measurements with the folin phenol reagent. *J Biol Chem* **193**: 265–269

McKeage MJ, Mistry P, Ward J, Boxall FE, Loh S, O'Neill C, Ellis P, Kelland LR, Morgan SE, Murrer BA, Santabarbara P, Harrap KR and Judson IR (1995) Phase 1 and pharmacological study of an oral platinum complex (JM216): dose-dependent pharmacokinetics with single dose administration. *Cancer Chemother Pharmacol* **36**: 451–458

McLlwrath AJ, Vasye PA, Ross GM and Brown B (1994) Cell cycle arrests and radiosensitivity of human tumour cell lines. Dependence on wild-type p53 for radiosensitivity. *Cancer Res* **54**: 3718–3722

Mellish KJ, Kelland LR and Harrap KR (1993) In vitro platinum drug sensitivity of human cervical squamous cell carcinoma cell lines with intrinsic and acquired resistance to cisplatin. *Br J Cancer* **68**: 240–250

Mellish KJ and Kelland LR (1994) Mechanisms of acquired resistance to the orally active platinum-based anticancer drug JM216 [bis acetato-ammine dichloro cyclohexylamine platinum (IV) in two human ovarian carcinoma cell lines. *Cancer Res* **54**: 6194–6200

Mistry P, Kelland LR, Abel G, Sidhar S and Harrap KR (1991) The relationships between glutathione, glutathione-S-transferase and cytotoxicity of platinum drugs and melphalan in eight human ovarian carcinoma cell lines. *Br J Cancer* **64**: 215–220

Oberhammer F, Wilson JW, Dive C, Morris ID, Hickman JA, Wakeling AE, Walker PR and Sikorska M (1993) Apoptotic death in epithelial cells, cleavage of DNA to 300 and/or 50 kb fragments prior to or in the absence of internucleosomal fragmentation. *EMBO J* **12**: 3679–3684

O'Neill CF, Orr RM, Kelland LR and Harrap KR (1995) Comparison of platinum binding to DNA and removal of total platinum adducts and inter-strand cross-links in three human ovarian carcinoma cell lines sensitive and resistant to cisplatin. *Cellular Pharmacol* **2**: 1–7

O'Neill CF, Ormerod MG, Robertson D, Titley JC, Cumber-Walsweer Y and Kelland LR (1996) Apoptotic and non apoptotic cell death induced by cis and trans analogues of a novel ammine(cyclohexylamine) dihydroxodichloroplatinum(IV) complex. *Br J Cancer* **74**: 1073–1045

Ormerod MG, Payne AWR and Watson JV (1987) Improved programme for the analysis of DNA histograms. *Cytometry* **8**: 637–641

Ormerod MG, O'Neill CF, Robertson D and Harrap KR (1994*a*) Cisplatin induces apoptosis in a human ovarian carcinoma cell line without concomitant internucleosomal degradation of DNA. *Exp Cell Res* **211**: 231–237

Ormerod MG, Orr RM and Peacock JH (1994*b*) The role of apoptosis in cell killing by cisplatin: a flow cytometric study. *Br J Cancer* **69**: 93–100

Ormerod M, O'Neill C, Robertson D, Kelland LR and Harrap KR (1996) Cis-diamminedichloroplatinum(II)-induced cell death through apoptosis in sensitive and resistant human ovarian carcinoma cell lines. *Cancer Chemother Pharmacol* **37**: 463–471

Perego P, Giarola M, Righetti SC, Supino R, Caserini C, Delia D, Pierotti MA, Miyashita T, Reed JC and Zunino F (1996) Association between cisplatin resistance and mutation of the p53 gene and reduced bax expression in ovarian carcinoma cell systems. *Cancer Res* **56**: 556–562

Pestell KE, Medlow CJ, Titley JC, Kelland LR and Walton MI (1998) Characterisation of the P53 status, Bcl-2 expression and radiation and platinum

 *© 1999 Cancer Research Campaign*

drug sensitivity of a panel of human ovary cancer cell lines. *Int J Cancer* **77**: 913–918

Petersen LN, Mamenta EL, Stevenser T, Chaney SG and Bohr VA (1996) Increased gene specific repair of cisplatin induced interstrand crosslinks in cisplatin resistant cell lines and studies on carrier ligand specificity. *Carcinogenesis* **17**: 2597–2602

Poon GK, Raynaud FI, Mistry P, Odell DE, Kelland LR, Harrap KR, Barnard CFJ and Murrer BA (1995) Metabolic studies of an orally active platinum anticancer drug by liquid chromatography-electrospray ionisation-mass spectrometry. *J Chromatogr* **712**: 61–66

Raynaud FI, Mistry P, Donaghue A, Poon GK, Kelland LR, Barnard CFJ, Murrer BA and Harrap KR (1996*a*) Biotransformation of the platinum drug JM216 following oral administration to cancer patients. *Cancer Chemother Pharmacol* **38**: 155–162

Raynaud FI, Odell DE and Kelland LR (1996*b*) Intracellular metabolism of the orally active platinum drug JM216: influences of glutathione levels. *Br J Cancer* **73**:

Sorenson CM and Eastman A (1988) Influence of cis-diamminedichloroplatinum (II) on DNA synthesis and cell cycle progression in excision repair proficient and deficient Chinese hamster ovary cells. *Cancer Res* **48**: 6703–6707

Twentyman PR, Wright KA, Mistry PA, Kelland LR and Murrer BA (1992) Sensitivity to novel platinum compounds in panels of human lung cancer cell lines with acquired and inherent resistance of cisplatin. *Cancer Res* **52**: 5674–5680

Vaisman A, Varchenko M, Said I and Chaney SG (1997) Cell cycle changes associated with the formation of Pt-DNA adducts in human ovarian carcinoma cells with different cisplatin sensitivity. *Cytometry* **27**: 54–64

Winters ZE, Ongkeko WM, Harris AL and Norbury CJ (1998) p53 regulates Cdc2 independently of inhibitory phosphorylation to reinforce radiation induced G2 arrest in human cells. *Oncogene* **17**: 673–684

Yaginuma Y and Westphal H (1992) Abnormal structure and expression of the p53 gene in human ovarian carcinoma cell lines. *Cancer Res* **52**: 4196–4199

Zaffaroni N, Silvestrini R, Orlandi L, Bearzatto A, Gornati D and Villa R (1998) The induction of apoptosis by taxol and cisplatin and effect on cell cycle-related proteins in cisplatin, sensitive and resistant human ovarian cancer cells. *Br J Cancer* **77**: 1378–1385

Zhen W, Link CJ, O'connor PM, Reed E, Parker R, Howell SB and Bohr VA (1992) Increased gene-specific repair of cisplatin intrastrand cross-links in cisplatin-resistant human ovarian cancer cell lines. *Mol Cell Biol* **12**: 3689–3698

*© 1999 Cancer Research Campaign*
